# Transforming food environments: a global lens on challenges and opportunities for achieving healthy and sustainable diets for all

**DOI:** 10.3389/fsufs.2024.1366878

**Published:** 2024-06-28

**Authors:** Ee Von Goh, Nafiisa Sobratee-Fajurally, Antonio Allegretti, Mallika Sardeshpande, Maysoun Mustafa, Susan Helen Azam-Ali, Rose Omari, Johanna Schott, Vimbayi Grace Petrova Chimonyo, Daniela Weible, George Mutalemwa, Tafadzwanashe Mabhaudhi, Festo Massawe

**Affiliations:** 1 School of Biosciences, Faculty of Science and Engineering, University of Nottingham Malaysia, Semenyih, Malaysia; 2 World Vegetable Center, Tainan, Taiwan; 3 Centre of Transformative Agricultural and Food Systems, University of KwaZulu-Natal, Durban, South Africa; 4 Institute for Natural Resources, Pietermaritzburg, South Africa; 5 Department of Sociology, St. Augustine University of Tanzania, Mwanza, Tanzania; 6 Lancaster Environment Centre, Lancaster University, Lancaster, United Kingdom; 7 Science and Technology Policy Research Institute, Council for Scientific and Industrial Research, Accra, Ghana; 8 Johann Heinrich von Thünen Institute, Braunschweig, Germany; 9 International Maize and Wheat Improvement Center (CIMMYT), Harare, Zimbabwe; 10 Centre on Climate Change and Planetary Health, London School of Hygiene and Tropical Medicine, London, United Kingdom

**Keywords:** global food environments, nutrition security, environmental sustainability, partnership for the goal, sustainable development goals, transdisciplinary research, participatory approach

## Abstract

Food environments are rapidly changing globally, both in developed and developing contexts, contributing to poor dietary habits and environmental concerns. As a result, more than 80% of countries in the world face different forms of malnutrition, while the environment faces further degradation due to unsustainable production and consumption patterns. Understanding food environments in diverse settings via a global lens is critical for facilitating the global transition to sustainable and healthy food environments. A virtual workshop was held with stakeholders from five nations (Germany, Ghana, Malaysia, South Africa, and Tanzania) representing varying levels of development to interrogate global food environment concerns and propose cross cutting thematic areas that may be explored and addressed through policy change and intervention. The workshop initiated a transdisciplinary project to shape food environments for sustainable and healthy diets. The Reference Manual for Convenors of Food Systems Summit Dialogues for United Nations Food Systems Summit (UNFSS) (United Nations, 2020) was used as guidance to ensure that an inclusive mix of stakeholders were invited. The stakeholders included key players from public and private sectors in disciplines of agriculture, agro-forestry, environment and ecology, education, food retail and market, trade and commerce, health care and nutrition. Following the workshop discussion, the findings were analyzed using a general inductive approach. Through triangulation of findings, we identified the common challenges and opportunities for achieving collective nutritional, social and environmental sustainability in the modern food environments, which have become more universal globally. It is evident that research and data are essential for sustainable development of food systems, while Sustainable Development Goal (SDG) 17 – Partnership for the Goals - should be placed at the core of the transformative process. We proposed several research-driven transdisciplinary interventions to facilitate a paradigm shift from the profit logic model over everything else, and to counter the existing policy fragmentation and systemic challenges to making food environments nutrition-sensitive and socially and environmentally sustainable.

## 1 Introduction

Food environments are the different contexts (physical, economic, political and socio-cultural) where people interact with the food systems (Downs et al., 2020). They are the points of convergence where global trends and processes meet people’s basic everyday activity of procuring and acquiring food for themselves, their families and communities (Herforth and Ahmed, 2015). Within food environments questions of availability, affordability, convenience and desirability of food eventually shape people’s diets and ultimately determine people’s health and nutritional outcomes as well as the sustainability of the environment and its resources (Willett et al., 2019).

Unhealthy food environments can contribute to malnutrition and nutrition insecurity through limited access, availability and affordability of nutritious food coupled with strong competition from nutrient poor, energy dense ultra-processed food. The triple burden of malnutrition (undernutrition, overweight/obesity, and micronutrient deficiency) is a food-related, global challenge that affects all nations regardless of socio-economic status and level of development: 88% (124 countries) experience more than one form of malnutrition, with 29% (41 countries) having high levels of all three forms (Global Nutrition Report, 2018). In 2021, with more than 42% of the world’s population unable to afford a healthy diet—an increase of 134 million people compared to 2019, before the pandemic—there is a concurrent rise in the consumption of processed and convenience foods due to accelerating urbanization, leading to increased rates of overweight and obesity across urban, peri-urban, and rural areas (FAO, 2023a). Unfortunately, most stakeholders pay little attention to poor diets, obesity and diet-related non-communicable diseases (NCDs) but rather focus strongly on undernutrition (Global Nutrition Report, 2022). In fact, trends of lower overweight in peri-urban areas and higher overweight in some rural areas compared to urban areas have been observed (FAO, 2023a).

Research conducted on food environments has been steadily growing in the last decade, expanding its geographical focus beyond the Global North, to low- and middle-income countries (Turner et al., 2018, 2020, 2022; Li et al., 2019; Constantinides et al., 2021; Kebede et al., 2022; Laar et al., 2022; Kumar et al., 2023). The growing attention paid to food environments, especially as opportunistic moments for policy interventions, has made it a rapidly evolving field of inquiry, with a florid debate around knowledge and methodological gaps. At the primary level, studies of food environments have traditionally been conducted at national and sub-national level (O’Meara et al., 2022) being grounded in the specific local geographies and landscapes of stores, restaurants and prices (McKinnon et al., 2009; Lytle and Sokol, 2017). Contextual knowledge of food environments, in particular the ‘lived experiences’ of people within them, is recognized as crucial for devising relevant policies (Miewald et al., 2010; Spires et al., 2021). More deeply, there is growing consensus that context specific research needs to be embedded in broader analyses at the global level to achieve a range of objectives at the intersection of environmental and human health (O’Meara et al., 2022). This recognition has led to the development of analytical tools for monitoring and benchmarking food environments globally by tailoring monitoring tools to the local context (Vandevijvere and Swinburn, 2014; Downs et al., 2020; Mann et al., 2021; Sacks et al., 2021).

The merging of these different scales in the analysis of food environments is the first contribution of this paper. In March 2021, the authors, as members of a multinational consortium of researchers from Germany, Ghana, Malaysia, South Africa and Tanzania, organized a virtual stakeholder workshop entitled “Food environments: a shared understanding.” The consortium represents five countries at different stages of development and with complex food environments embedded in the respective food systems (refer to Appendix 1 for visualization of the countries’ progress in selected key indicators that span across the spectrum). The overall objective of the workshop was to interrogate issues of food environments in the participating consortium countries and highlight areas that could be researched and addressed through global policy change and intervention. An intended outcome was to develop a global lens through which to analyze food environments. In addition, the workshop served as Phase 0 for a shared understanding of food environments across the five countries for development of a transnational, multi-disciplinary research proposal. Phase 0 is also known as the pre-launch phase for transdisciplinary projects, beginning before problem framing and research co-design, and is crucial to unleashing the full transformative potential of transdisciplinary research (Horcea-Milcu et al., 2022).

The second contribution of this paper is the application of sustainability as an analytical framework to interpret workshop findings and build a comprehensive global perspective on food environments. As Downs et al. (2020) argue, food environment research lags behind with respect to incorporating sustainability as an indicator for evaluating food environments. Food sustainability is multi-faceted and multi-dimensional as it incorporates economic, environmental, and social aspects (Berry et al., 2015; Rapinski et al., 2023) while addressing challenges around production, consumption, and equity in food systems (Garnett, 2013; Medina and Sole-Sedeno, 2023). Addressing food sustainability is integral towards finding solutions to the diet-environment-health sustainability ‘trilemma’ (Tilman and Clark, 2014). Because of the interlinkages of these challenges, cross-sectoral and cross-regional analyses are crucial (Rapinski et al., 2023).

We address these complexities around sustainability by mapping the workshop findings against the 17 Sustainable Development Goals (SDGs). This approach aims to highlight the relevance of the concept of ‘food environment’ in global development debates, and policy as an analytical lens through which to address food-related issues. The paper explores the common challenges and opportunities for achieving nutritional, social and environmental sustainability in global food environments. This approach is original and highlights the urgency of addressing food-related questions within the context of food systems, rather than a piecemeal approach, and the development of effective strategies through intersectoral policies (Medina and Sole-Sedeno, 2023).

The significance of this holistic and global approach to the analysis of food environments across a spectrum of different regions, reinforces the need to consider various sectors and spheres (Medina and Sole-Sedeno, 2023), and to involve multiple stakeholders in food policy dialogue (Spires et al., 2023). This approach will aid policies and decision-making towards the promotion of vital socio-cultural outcomes including nutrition and health, cultural and heritage, labor conditions, and animal welfare (FAO, 2018), and for consumers to make healthier and (socially and environmentally) sustainable food choices (Downs et al., 2020).

## 2 Methods

**Figure 1:**
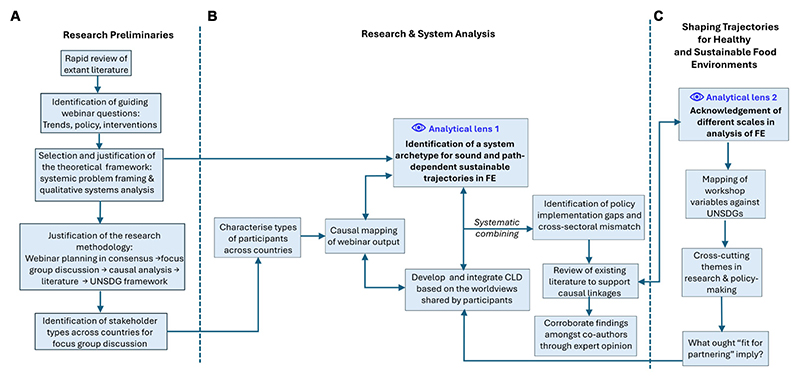
Research protocol **(A)** Research preliminaries for virtual workshop. **(B)** Research and System Analysis. Analytical lens 1 emerges as the dominant form of inquiry to triangulate the causal loop analysis. **(C)** Mapping of Analytical lens 2 recognizes the need to consider cross-cutting issues at the research (local to landscape research impact) and policy (cross governance level coherence) interface. What should ‘fit for partnering’ entail? This concept not only denotes suitability for collaboration but also encompasses the objective of critically examining issues related to food environments in the participating consortium countries. It aims to highlight areas that could be researched and addressed through global policy change and intervention. CLD, causal loop diagramming; FE, food environment, UNSDG, United Nations Sustainable Development Goals Double-sided arrows indicate triangulation. Double arrows indicate verification through triangulation.

The workshop was carried out using an online platform to enable access from all representative stakeholders in the five countries. A three-staged approach was used: (i) pre-workshop preparation to identify key emergent issues for interrogation and targeting invitees, (ii) administering the workshop and driving the discussion, (iii) de-briefing and data analysis. Figure 1 describes the steps involved in development of the methods. Three main parts are highlighted: (A) the research preliminaries for the virtual workshop preparation, (B) the core research steps, i.e., literature research and systems analysis, and (C) the systems analysis and UNSDG framework where the transformative aspects are examined in detail. Figure 1 also highlights the triangulation of methods to scrutinize the data - the systemic analysis, corroborated with the scholarly literature and embedded within the framework of the UNSDGs. Two analytical lenses emerged from the approach, namely the use of sustainability as a framework to interrogate science-policy interface in food environments and the cross-scale interactions across food environment dimensions.

### 2.1 Pre-workshop preparation

#### 2.1.1 Identification of key topics and questions

Pre-workshop preparation involved a rapid review of literature on three focal points – trends, policy, and interventions (see Appendix 2 for a summary table). Synthesis of the literature involved transposing these three elements across countries to formulate three emergent key questions: (1) How can food environments move beyond emphasizing food security to encouraging nutritional security? (2) How can environmental and social sustainability be incorporated into food environments? and (3) How can the gap between policy and execution be bridged to ensure sustainable healthy food environments? The workshop discussion was divided into three sessions, with each key question being discussed in each session. All invitees were invited to participate in every session.

#### 2.1.2 Identification and invitation of discussants

The Reference Manual for Convenors of Food Systems Summit Dialogues for United Nations Food Systems Summit (UNFSS) (United Nations, 2020) was used as guidance to ensure that an inclusive mix of stakeholders from the focal countries were invited. A systematic approach was adopted to ensure representation from diverse backgrounds and perspectives relevant to the study’s objectives. Potential participants were identified through expert networks and recommendations from key informants in each focal country, prioritizing individuals with expertise in areas delineated by the UNFSS guidelines (United Nations, 2020). Over 90 people registered for the workshop, and 75 attended. Participants from various countries (Germany, Ghana, Malaysia, South Africa, Tanzania and the United Kingdom) represented a variety of stakeholder groups (including government, universities, private consulting firms, and NGOs) and sectors (including agriculture, agro-forestry, environment and ecology, education, food retail and market, trade and commerce, health care and nutrition). Each participant brought valuable insights and expertise to the workshop, contributing to the comprehensive exploration of food environment complexities in the focal countries as well as providing a global overview. Their diverse backgrounds and perspectives enriched the dialogue, leading to the formulation of research recommendations aimed at addressing these complexities.

### 2.2 Administration of the workshop

Administration of the workshop was planned and executed to ensure a conducive environment for open dialogue and meaningful participation. Each session was facilitated by an experienced moderator who was selected and briefed to foster an atmosphere of respect and trust among participants. Drawing from recommendations by O’Haire et al. (2011) and Heywood et al. (2021), moderators were tasked with creating a safe space where all discussants felt comfortable contributing their individual perspectives. This involved measures such as seeking consent for video recording, transcription, and use of the discussion for analysis, while also avoiding any biases.

Moderators were responsible for ensuring equitable participation, crafting key questions for discussion, maintaining a neutral demeanor, and summarizing discussions to reflect diverse opinions fairly. They encouraged active engagement by inviting participants to raise virtual hands or contribute to the live chat box, thereby facilitating a dynamic exchange of ideas. Additionally, moderators provided contact details for follow-up discussions, ensuring that any remaining questions or comments could be addressed after the workshop concluded.

Guided by Krueger’s (2000) categories, the discussion sessions were structured around carefully crafted guiding questions to elicit comprehensive insights from participants. To promote openness and information sharing, comments made during the workshop were not attributed to individual discussants. The workshop spanned a total of 3 h, allowing ample time for in-depth exploration of the topic and robust exchange of perspectives among participants. Through these deliberate measures, the workshop administration aimed to maximize the effectiveness of the discussions and ultimately generate valuable insights for addressing the complexities of food environments.

### 2.3 Problem framing and data analysis

Data analysis involved several steps to ensure a comprehensive understanding of the workshop discussions and to frame key issues related to food environments. Stage one was the transcription of the recordings of the workshop discussions to capture all nuances and insights shared by participants. These transcripts served as the primary data source for the analysis.

Using a general inductive approach (Thomas, 2006), the workshop findings were analyzed to identify categories and themes emerging from the discussions. For each theme, categories such as sub-themes, issues/problems, and challenges/bottlenecks were identified. These categories were subsequently mapped against the relevant United Nations Sustainable Development Goals (UNSDGs) to frame the problem statement and understand the challenges highlighted by stakeholder engagement.

For quality control, to reduce bias and ensure the reliability of the data interpretation, the transcripts were analyzed by two independent groups. The preliminary analyses carried out by one group were cross-checked by the other group to ensure the accuracy and trustworthiness of the qualitative data. Additionally, the results were triangulated with a literature review and local expert opinion to corroborate the findings and ensure a robust analysis.

Findings were mapped against and organized according to relevant SDGs and respective targets. By framing our analysis around the SDGs, this paper intends to keep the analysis abreast of current sustainability debate (Hák and Moldan, 2016; Mainali et al., 2018; Wood et al., 2023). Also, by mapping the themes against other goals, beyond the goal of Zero Hunger (Goal 2), in accordance with other research (Gil et al., 2019; Bizikova et al., 2020; Ghosh-Jerath et al., 2020; Mensi and Udenigwe, 2021; Vogliano et al., 2021), we are able to consider a more holistic and broader concept of sustainability in food environments. The mapping included consideration of education (Goal 4), peace (Goal 16), urbanization (Goal 11), and overall consumption patterns beyond food (Goal 12), and how the interaction of these elements are gender sensitive (Goal 5), and impact inequalities (Goal 10). This allows us to bring out the links between food and different indicators of wellbeing beyond nutritional outcomes while considering the different social, cultural and ecological dimensions of sustainability (Downs et al., 2020; Fanzo and Davis, 2021).

A realist approach (Hewitt et al., 2012) was used to frame the elements shaping the food environment across the countries discussed in the workshop. Qualitative system dynamics models, including causal loop diagramming (CLD) and stock accumulation, were used to illustrate the interlinkages across domains and scales. These models provided a conceptual framework to understand the dynamics of the food environment system and how various factors interact to influence outcomes over time.

Local–global goals were highlighted by applying the relevant UN SDGs. Diagrammatic representations of relevant subsystems are shown in the form of causal loop diagramming (CLD) and stock accumulation in a qualitative portrayal of the dynamics of the food environment system across the countries. Conceptually, stocks are entities or variables that can be accumulated or depleted. The flows capture the activity related to stock (Sterman, 1989, 2010; Sweeney and Sterman, 2000). In the CLD, arrows show the influence of one variable on another—a change in the cause leads to a change in the effect. The polarity of the arrows (A → + B or A → - B) indicates the factual relationship between any two nodes, which illustrates the causal link (Morecroft, 2020). A balancing loop is a cycle in which the effect of a variation in any variable propagates through the loop and returns to the variable a deviation opposite to the initial one (i.e., if a variable increases in a balancing loop, the effect through the cycle will cause a decrease to the same variable and vice versa). In contrast, a reinforcing loop is a cycle in which the effect of a variation in any variable propagates through the loop and returns to reinforce the initial deviation (i.e., if a variable increases in a reinforcing loop, the effect through the cycle will result in an increase to the same variable and vice versa).

Simple stock and flow networks were used to describe accumulation, and the corresponding rate of change over time. In trying to understand a particular ‘system of interest’, the interplay of balancing and reinforcing loops gives rise to a realistic multi-loop system that explains behavior through time (Morecroft, 2020). In this paper, the system of interest refers to how, during the workshop, participants (i) assessed the forces governing food environments in Germany, Malaysia, Ghana, South Africa and Tanzania and (ii) proposed possible evidence-based approaches and implementation mechanisms to inform policymaking. Through this analysis, the authors gained insights into the complex dynamics of food environments which allowed them to recommend effective strategies to address the challenges.

## 3 Findings – the analysis of causality across domains and scales in food environments

**Figure 2:**
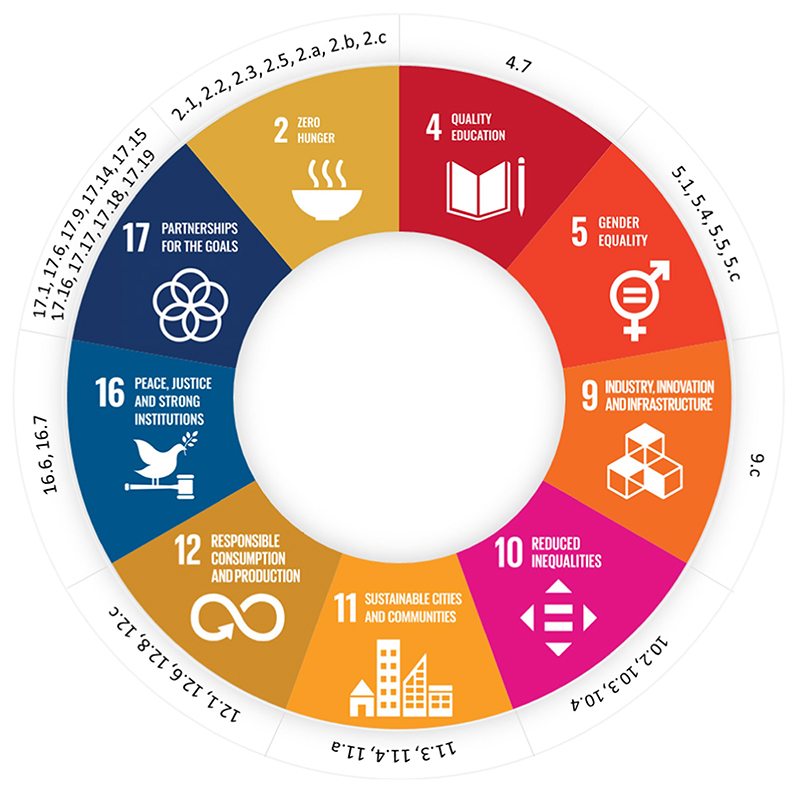
SDG Targets relevant to the workshop outcomes. * The SDG target descriptions are shown in Appendix 3.

Overall, the workshop discussions span 9 SDGs and 34 Targets (T) (Figure 2) and reflect the interrelated nature of food environments’ multiple facets and dimensions. The consensus that emerged from the workshop is the bi-directional relationship of individuals with the food environment; the food environment nudges consumer behavior, while at the same time, it is shaped by food culture and consumer preferences. Every individual has complex interactions with their own food environment—the point at which they engage with the food system—and these are influenced by individual, household and organizational factors. Agency and one’s capacity to make food choices depend on many external factors. The common issues, challenges as well as areas of opportunity identified during the workshop, which impact food behavior and consumption patterns, can be summarized under the following broad categories: Policies; Individual agency; Urbanization; and Sustainable consumption. These themes will be explored further in the following subsections, illustrating how they collectively address the three key questions raised during the workshop on promoting nutritional security, incorporating sustainability, and bridging the policy-execution gap in food environments.

### 3.1 Cross-sectoral policy mismatch, lack of understanding and misplaced priorities

There was an overwhelming consensus among participants that the conditions surrounding food production, distribution, and marketing tend to favor nutrient-poor processed (NPP) foods over fresh and nutritious foods such as fruit and vegetables. The reasons behind this are numerous and multi-faceted, not being ascribed to one single root cause; what people eat depends on the food environment in which they live, rather than simply personal choices or preferences. The overriding opinion of most participants was that efforts to reverse the situation or provide alternatives (to NPP), such as public policies or other food-related initiatives in the fields of advertising, promotion, and distribution, lag behind the expected levels.

According to stakeholders, an important root cause of the current state of affairs is the fundamental disconnect between the overall apparatus of production/distribution of NPP and national governments, which are vested with the important public function of improving the nutritional outcomes of their populations. Governments often intertwine food policies primarily with agricultural policies (T 17.4), overlooking the interconnectedness between food, agriculture, environment, and health. This oversight neglects crucial aspects such as awareness raising and health promotion, failing to address the broader environmental implications of food production and consumption. Consequently, food policies rarely tackle key issues such as sustainability awareness raising and health promotion (T 2.2, 17.10, 17.14). This is true of countries in the lower income range, such as Tanzania, but also middle-income countries, such as Malaysia. Historical circumstances were acknowledged to have led to the current situation; these are connected to the boom in the food industry that has dramatically improved the availability of NPP, but also current circumstances in terms of governments abiding by the ‘freedom of choice’ (T 11.4, 17.14, 17.15) doctrine, according to which governments should not interfere with market drivers.

It was asserted that the governments’ quantity-based (food security) approach when prioritizing the availability of food (often through ‘cheap’ calories) over nutrients for better health (nutritional security) in the population has facilitated the growing power of private players in the food industry; these have at their disposal increasingly more resources and bigger budgets to enhance their market and distribution networks (T 2.1, 2.2, 17.10, 17.14) as well as, importantly, advertising via traditional media and social media tools through which they promote their products. According to the participants, this growth has allowed the large food industry to beat the market competition as they can afford to market their products at lower prices, with a large margin for profit from processing, in comparison to fresh foods with shorter shelf life and higher production costs (T 2.2, 2c, 4.7). The distribution network for processed food is extensive and well-developed, penetrating the poorest and most remote areas, making these foods more readily available. Concurrently, on the one hand, governments have been unable to make a dent in food advertising (particularly through social media), which lacks transparency (T 2.2–17.14), and on the other, they have not been able to support the establishment of equally strong and pervasive marketing and distribution networks and infrastructures for healthier foods to counterbalance the negative nutritional outcomes (overweight, obesity) that derive from consumption of NPP (T 2.2, 2a, 2b). Unsurprisingly, the poorer sectors of societies and communities, such as the urban poor, including those in developed nations, take the brunt of this situation, ultimately consuming NPPs which are more affordable and more readily available in areas serving disadvantaged communities (T 2.1, 2.2, 2c, 4.7, 17.10, 17.14). This phenomenon aligns with the findings of de Bruin and Holleman (2023), as documented in a recent FAO Agricultural Development Economics Working Paper.

An important policy-related consideration that workshop participants agreed on was that nutrition security issues should not be addressed solely within the arena of health but also within the arena of agriculture, food industry and related policies, hence making nutritional outcomes a cross-cutting objective among interdependent policy sectors (T 2.2, 17.9, 17.16). Localizing the food supply chain with more direct farm-to-consumer links was proposed as a possible direction towards this objective as it would make healthy foods more accessible to disadvantaged communities (T 2.3, 2c). All in all, policy integration emerged as an important challenge within the policymaking arena. A food environment approach can potentially break the business-as-usual approach in policy making and implementation that, to date, has historically been done in (policy) silos.

### 3.2 Consumer responsibility narrative that largely ignores structural issues that make unhealthy and unsustainable diets an easier and cheaper option

The second key area of focus was the crucial question of food choices, the personal agency in making them, and how these are approached in education for behavioral change. The consensus opinion was that a focus on food ‘choices’ or ‘preferences’ as an individual exercise of agency for behavioral change might not be very effective without a holistic approach that acts upon the overall food environment (T 4.7, 17.14) and the broader food system. It is crucial to consider the broader global food system and the structures that shape supply chains, such as trade policies, profit and power dynamics, and the influence of large food corporations. These factors significantly impact food availability and affordability, often making cheaper, unhealthy food options more accessible. The pervasiveness and ease of access to unhealthy, ultra processed, snacking and packaged foods has led to an ‘acquired taste’ for NPP across the socioeconomic spectrum and development range of countries (T 4.7, 12.1, 12.8).

The prevalent consumer responsibility narrative tends to overlook these structural issues, which can undermine efforts to promote sustainable and healthy diets. Hence, any possible interventions must be tailored to the specific socio-cultural and economic context (i.e., the food environment) rather than targeting individuals. Nutrition education imparted to children and adolescents may provide useful information. However, it often fails to impact on individual agency to make better food-related choices regarding sustainable and healthy foods, as the intervention programs omit to take into account the wider context (T 4.7, 12.8). Focusing on individual choices in terms of food preferences while overlooking the structures around which food availability and consumption are built can undermine individuals’ true agency in choosing what is good for them, and ultimately often limits the effectiveness of interventions in behavioral modification (T 4.7, 17.14).

The discussion emphasized the influence of gender, traditional values, poverty, education, and socio-economic class on individual agency. It became evident that individual agency alone is insufficient to drive meaningful change. Instead, a collective responsibility is needed to address the structural issues ingrained within the global food system. By recognizing the influence of socio-cultural and economic factors on food environments and individual agency, we pave the way for inclusive interventions tailored to diverse contexts. Through shared responsibility, encompassing education, awareness, and structural reform, we can foster environments that empower individuals to make sustainable and healthy food choices, thus driving positive shifts towards a more equitable and nourishing food future.

### 3.3 Planning and policies that struggle to keep up with rising urban issues

There was general consensus that the lack of policies for inclusive urbanization has resulted in significant nutrition challenges. A widespread opinion among participants was that all countries under consideration here have similar challenges handling the influx of population to the urban areas. These include a shortage of job opportunities and settlements that are not conducive for cooking or home-preparation of food (T 10.2, 10.3, 10.4, 11.a). The urban poor suffer the greatest nutritional impact, having insufficient resources to make nutritious food choices, and often rely heavily on NPP and street food (T 2.2, 2c, 4.7). Unemployment has pushed people, to some extent, into informal food businesses, and more so during the Covid-19 pandemic (T 11.3, 11.a). However, little is known about the informal food sector and its nutritional role across the urban gradient (T 11.3, 11.a).

There was broad agreement that the diversity of cultural heritage in relation to urbanization is often overlooked (T 11.4). Urbanization has inevitably caused nutrition transition and homogenization of the diet, thus reducing the biocultural diversity of food consumed. The shrinking diversity of food plates is also linked to significant environmental implications, such as soil nutrient depletion that occurs due to increased monocropping (T 11.4, 11.a) as part of industrial agriculture. The new urban food landscape also means that suppliers in urban markets sometimes source globally for seasonal products, which entails high economic and environmental costs. There is a lack of understanding of how these supply and demand processes impact the global socio-ecological cost.

Moreover, current policies are skewed towards pro-urbanization trends where the built environment essentially promotes the individualistic lifestyle that impacts food choice, availability and aspirations. Over-reliance on processed and/or convenience foods has led to the erosion of the food’s cultural heritage and loss of bio-cultural diversity of the food plate. Alongside negative food-related effects of urbanization, there are trends in recreating ‘traditional’ food cultures and traditions within urban settings. These trends offer opportunities to create diversity within urban food environments and to enrich them via promotion and increased uptake of traditional foods. To fully leverage these opportunities, participants raised several pertinent questions: how do we keep traditional knowledge and foods alive in an increasingly cosmopolitan and globalized world? What does it take for people to recreate their food cultures in urban settings? (T 11.4, 17.6, 17.9).

Summarily, navigating the complexities outlined above, where planning and policies struggle to keep pace with escalating urban issues, necessitates collective action towards inclusivity, cultural preservation, and environmental sustainability in urban settings, fostering awareness and appreciation for diverse food cultures, mitigating the ecological impact of urbanization, and ultimately bolstering the resilience of urban communities.

### 3.4 Responsible consumption and production – the dilemma between individual and systemic change

The topic of responsible consumption and production created lively debate, particularly on the dilemma of individual versus systemic changes. While consumers need relevant information and awareness for sustainable development and lifestyles in harmony with nature (T 12.8), participants questioned the applicability and limits of school education in providing this information and awareness (T 4.7, 12.8). There were also issues around the reluctance of stakeholders to change in response to abstract research recommendations (T 4.7, 12.8), in addition to the difficulty in overcoming acquired preferences for NPP foods and affordability and accessibility issues, as discussed in the earlier subsections. While recognizing that governments are generally reluctant to tax and incentivize food for sustainability, it was suggested that governments could and should take the lead by devising policies to internalize externalities (social, ecological, environmental, public health) into food prices (T 12.1, 12.6, 12.c, 17.10). This allows “fair” competition between NPP foods and healthy fresh food. However, one question that was raised and remained unanswered was who should bear the cost for “freedom of choice’. There were further discussions on whether the ‘true cost’ of foods would be borne by consumers primarily and concerns that consumers in the Global South could disproportionately be affected. Additionally, the effectiveness of displaying the true costs of food in influencing behavioral change is not well-known, and this practice may only appeal to privileged consumers who can afford to choose.

It was overwhelmingly agreed that responsibility for sustainable consumption and production should be shared at all levels (T 12.1, 17.10). Education programmers should not just focus on consumers but also target supply chain actors (T 12.8). Civil society and food policy councils can lead in localizing or regionalizing food supply (T 12.8, 17.14). Civil society groups can also contribute by educating and advertising ‘value’ versus ‘price’ for local and responsibly grown food (T 12.8, 17.14). Traditional media outlets and social media can be useful tools in nudging consumer behavior through localized content, along with local community mobilization by researchers and ministries of health (T 12.8, 17.14).

**Figure 3:**
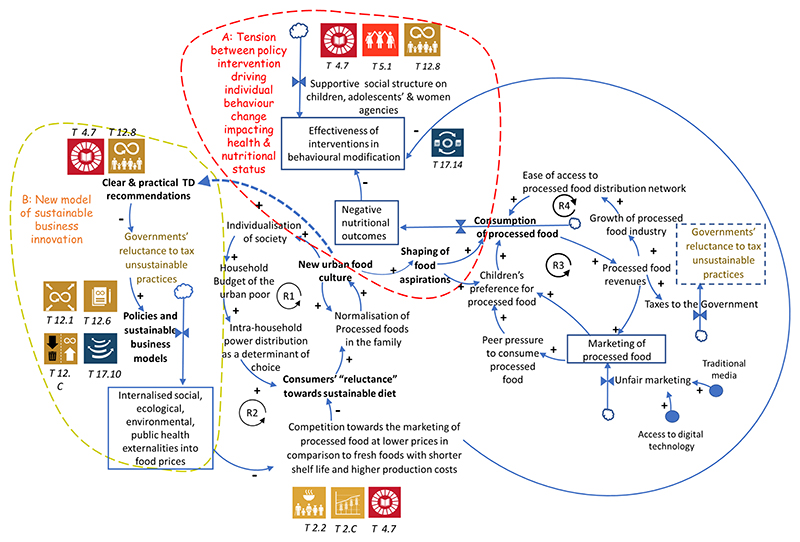
Systegram showing two emerging subsystems driven by the new urban food culture that shapes food aspirations. Subsystem A: The tension between policy interventions on individual behavior change impacting health and nutritional status versus the effect of systemic impediments that drive the consumption of processed foods. The effectiveness of interventions in behavioral modification is counteracted by marketing and advertisements that promote consumption that result in negative health outcomes. Subsystem B: New business model that ought to internalize social, ecological, environmental and public health externalities into food prices. The pre-requisite for Subsystem B is to materialize sufficient transdisciplinary and trans-boundary knowledge to understand the drivers and constraints emerging from the urban food culture with respect to processed foods. Vicious reinforcing loop, R1: Effect of individualization on the food choice determinants of the urban poor in developing countries. Virtuous reinforcing loop R2: This a solution-oriented loop showing how to internalize sustainability dimensions in food prices that ought to dampen the effect of normalization of processed foods at the household level (R2 loop: Competition towards the marketing of processed food at lower prices in comparison to fresh foods with shorter shelf life and higher production costs → Consumers’ “reluctance” towards sustainable diet→ Normalization of processed foods in the family→ New urban food culture→ Clear & practical transdisciplinary research recommendations→ Governments’ reluctance to tax unsustainable practices→ Policies and sustainable business models→ Internalized social, ecological, environmental, public health externalities into food prices→ Competition towards the marketing of processed food at lower prices in comparison to fresh foods with shorter shelf life and higher production costs). Loop R3: A vicious reinforcing loop where marketing influences consumption of NPP and hence boost revenues. Similarly, in loop R4, the increased revenues enable the growth of the NPP industry that widens its distribution network to become easily accessible to consumers.

The findings indicate that the issues observed across food systems are highly interrelated. Individuals’ conflicts with the food environment are contrasted with systemic conflicts involving conditions, practices, or values affecting broader populations. Such systemic conflicts are rarely fully understood or addressed within interventions aimed at resolving individual conflicts to achieve sustainable healthy diets. The underlying sources of these conflicts, as depicted in Figures 2, 3, are connected to broader goals influencing how the problems can be addressed. Interventions targeting immediate individual conflicts often overlook systemic actors, perpetuating dysfunction in the personal food environment. Specifically, Figure 3 illustrates that the marketing competition favoring processed foods over fresh foods with shorter shelf life and higher production costs impacts the effectiveness of behavioral interventions. According to systems thinking principles, upstream causes may have downstream consequences spread across time, space, and scale (Morecroft, 2020). The workshop discussions further unpacked this causality pathway, demonstrating how factors such as reluctance for sustainable diets, normalization of processed foods in families, and new urban food culture drive unhealthy food consumption, leading to negative nutritional outcomes.

**Figure 4:**
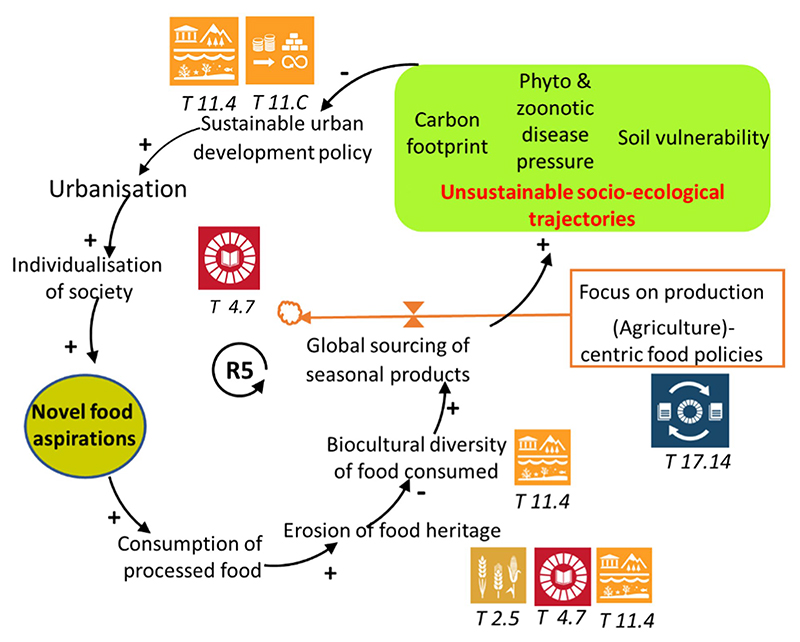
The environmental sustainability dimension as illustrated by the workshop participants. The consumption of processed food reduces the biocultural diversity of the food plate and increases global sourcing of seasonal products. Three negative consequences capable of jeopardizing sustainable outcomes were highlighted: Impact on carbon footprint, on plant/zoonotic disease pressure and on soil vulnerability. The implementation of sustainable urban policies ought to address the effects of urbanization on food aspirations and consumptions. Vicious negative reinforcing loop R5: Consumption of processed food → Erosion of food heritage → Biocultural diversity of food consumed → Global sourcing of seasonal products → Carbon footprint → Sustainable urban development policy → Urbanization → Individualization of society → Novel food aspirations → Consumption of processed food.

Consequently, the effectiveness of interventions in behavioral modification to promote healthy and sustainable diets is curtailed. Understanding the relationship between these variables in the real world requires transdisciplinary collaboration across different socio-technical and socio-ecological scales and a rethinking of new sustainable business models. Figure 4 encapsulates participants’ views on the counterintuitive effects of agricultural production-centric policies, highlighting how the emphasis on yield per hectare as a measure of national food security and export success, alongside the global sourcing of seasonal products, has increased the carbon footprint, thereby misaligning with sustainable urban development policies. These figures collectively contextualize the workshop discussion outcomes, highlighting the need for comprehensive, multi-faceted approaches to policy improvement by integrating robust data, fostering shared responsibility among stakeholders, and prioritizing comprehensive awareness-raising.

## 4 Discussion: shaping trajectories for healthy and sustainable food environments

### 4.1 Key cross-cutting themes – pillars for change

Considering the findings outlined in Section 3, there are three key cross-cutting themes that are crucial as arenas of intervention for better food environments: (i) inclusivity, (ii) education and awareness, and (iii) shared responsibility. There is a significant interconnection between these themes, which indicates the need for an integrated approach to achieve a sustainable transformation of food environments.

The relationship between food environments and food systems, both inter- and intra-countries, underscores the importance of shared responsibilities in addressing the complexities of the food environment and food systems, both within and between countries. Internationally, food systems are deeply interconnected through trade, global supply chains, and shared environmental resources. Decisions made by one country regarding food production, distribution, or consumption can significantly impact other nations, emphasizing the need for cross-border cooperation to tackle transboundary challenges such as nutrition security and environmental sustainability. This highlights the shared responsibilities between nations to develop a cohesive global framework and guidelines, ensuring consistent standards from nutritional regulations to sustainable urbanization. Existing frameworks, such as the New Urban Agenda (UN-Habitat, 2022) and the Milan Urban Food Policy Pact (MUFPP, 2015), provide a foundation but require harmonization and stronger global commitment. Such frameworks and guidelines are ineffective if not widely adopted and implemented, underscoring the necessity for international collaboration and dedication.

Within countries, food systems operate within diverse contexts that influences individuals’ access to nutritious and sustainable foods and dietary choices. Disparities persist across various dimensions such as socioeconomic status, geographical location, gender, and age. These intra-country dynamics significantly influence food behaviors and consumption patterns within households and communities. Interventions must account for these disparities to ensure equitable access to healthy food environments and dietary practices. To address these issues comprehensively, intervention programs must involve all sectors of society, shifting focus from individuals to communities and social intersections in decision-making processes. This requires understanding the environmental factors influencing individuals’ food-related choices, both within and across national borders, and considering personal experiences within food environments. Nutrition education programs should adopt an inclusive approach that acknowledges cultural heritage and societal identities, recognizing inequalities at all levels and dimensions and their compounding impacts on food behavior and consumption patterns (James, 2004; Atoloye et al., 2021). Empowering individuals through nutrition education programs is crucial, necessitating an understanding of the dynamics influencing decision-making within households or communities.

Collaborative efforts with stakeholders and various actors within these environments are equally crucial to disrupt ingrained consumer patterns, particularly in areas where unhealthy food choices are prevalent (Vogel et al., 2019; Livingstone et al., 2023; Vogel et al., 2023). Educating all actors becomes paramount, shaping expectations, understanding, beliefs, and the capacity to adopt recommended changes. Understanding the interconnectedness of various factors within food systems and adopting a systems thinking approach can help stakeholders comprehend the complexity of the issues at hand. By educating stakeholders about food systems, including the interplay between environmental, social, economic, and cultural factors, they can develop a more comprehensive understanding of the challenges and potential solutions. Moreover, promoting systems thinking can facilitate a shift in mindset from linear, isolated approaches to holistic, integrated strategies. This mindset shift is essential for stakeholders to recognize their roles within the broader food system and understand how their actions can influence outcomes at various levels. It can also help stakeholders identify leverage points for intervention and anticipate potential unintended consequences of their decisions. However, securing buy-in, particularly from the commercial sector actors, poses challenges due to prioritization of the bottom line and concerns about disruptions or financial risks. Overcoming skepticism requires building trust, demonstrating long-term advantages, and navigating diverse stakeholders. Effective communication and showcasing positive impacts on both business and societal goals are essential. Achieving these goals necessitates significant investment in robust structures for capacity building, technology access, data utilization, and resource mobilization.

In addressing the integration of inclusivity, shared responsibility, and education, it’s essential to incorporate governance elements and policing mechanisms to ensure fairness and accountability (Bui et al., 2019; Fanzo et al., 2021). Clear delineation of roles and responsibilities, as well as oversight mechanisms, is necessary to prevent inequities and ensure that interventions are implemented effectively (Guijt et al., 2021). This requires collaborative governance structures that involve stakeholders from diverse sectors and levels of society, fostering transparency, accountability, and trust (Guijt et al., 2021; Nikolaidou et al., 2023). Moreover, while empowering consumers with knowledge and skills is crucial, it must be complemented by structural changes that facilitate healthy and sustainable choices. Structural issues such as food availability, affordability, and marketing practices significantly influence consumer behavior, often overshadowing individual responsibility (Chen and Yang, 2014; Cooksey-Stowers et al., 2017; Constantinides et al., 2021). Hence, interventions should aim to reshape food environments by addressing these structural determinants, promoting equitable access to nutritious foods, and incentivizing responsible production and consumption practices. By aligning production and consumption practices with nutrition and sustainability goals and promoting cross-sectoral collaboration, stakeholders can pave the way for a more robust policy environment that better supports these crucial objectives, which will be discussed in detail in the following sub-section.

### 4.2 Current policy environments are not fit for purpose

Countries across the world, regardless of their development status, exhibit varying levels of policy incoherence nationally regarding agriculture innovation, productivity, and sustainability (OECD, 2019). Incoherence within strategic policy objectives perpetuates divergent policies, hindering cohesive progress, while inconsistencies among agriculture, innovation, and environmental policies undermine effectiveness and lead to unintended consequences (OECD, 2019). Many government policies and interventions persist in a top-down approach, resulting in fragmented implementation and impact across various sectors (Khan, 2021). Within the food system, different components are often overseen by separate local authorities, operating independently, leading to fragmented decision-making and potentially contradictory actions (Filippini et al., 2019). Despite the growing recognition of the importance of cross-cutting themes in shaping food environments, current policies often fall short in effectively addressing these complexities, both at the intra and inter-country levels. For instance, piecemeal policies that focus solely on specific crops or limited regions, can have a significant impact on the food environment. When policies are narrowly tailored to certain crops or regions, it can lead to imbalances in food production and distribution and reliance on food imports, which can be risky, as we have witnessed from the impacts of the COVID-19 pandemic, climate change, and conflicts (Paudel et al., 2023). This imbalance may result in limited access to diverse and nutritious food options. Additionally, when policies prioritize productivity without considering sustainability, they may inadvertently perpetuate environmental degradation and undermine long-term planetary health (Canavan et al., 2017; Benton and Bailey, 2019).

Urban food policies have emerged in response to the need to reorganize urban strategies due to the rising global urban population and rapid globalization fueled by market liberalization over the past three decades (FAO, 2015). The betterment of food environments requires innovative policy approaches that vary in their frameworks and must account for the unique themes and concerns emerging in local contexts, while enhancing coordination among diverse food system components and actors. This necessitates a stronger conceptualization of the urban, a clearer definition and articulation of the nature of governance and policy, and a more engaged focus on issues of power and inequities (Moragues-Faus and Battersby, 2021). Effective urban food policies require cooperation between private and public stakeholders to bolster the effectiveness of bottom-up initiatives from the private sector and top-down interventions from institutions (Filippini et al., 2019). However, a significant obstacle to ensuring inclusivity lies in the limited presence or absence of resources within consumer-focused civil society or grassroots organizations. This lack of resources hinders their ability to lead or actively participate in initiatives aimed at overcoming various challenges, such as the inertia associated with low food literacy, which is crucial for triggering meaningful change (Begley et al., 2020).

At the inter-country and regional levels, examples such as the European Union’s (EU) Common Agricultural Policy (CAP) showcase how policy frameworks aimed at promoting agricultural productivity often overlook social and environmental dimensions, contributing to inequalities in access to nutritious foods. The CAP is a significant policy framework aimed at promoting agricultural productivity within EU member states. However, several studies have highlighted how the CAP often overlooks important social and environmental dimensions. Scown et al. (2020) found that the distribution of CAP payments exhibits strong biases, with a disproportionate amount allocated to supporting viable farm income, while objectives related to environmental care, preserving biodiversity, and supporting vibrant rural areas receive significantly less funding. Moreover, Garcia-Bernardo et al. (2021) observed that payment inequality among CAP beneficiaries differs among member states, with new member states experiencing higher levels of inequality. Furthermore, the CAP’s payment schemes and market organization rules incentivize producers to prioritize certain types of agricultural production, particularly fruits and vegetables, which receive disproportionately low funding compared to sugar and livestock products (Pushkarev, 2015) influencing the overall food environment in Europe (Pushkarev, 2015; Erklavec et al., 2021). This has implications for dietary choices and health outcomes, as evidenced by research linking CAP budgetary priorities to changes in diets and the prevalence of obesity (Pushkarev, 2015). Similar supranational institutions do exist in other regions, such as the African Union, but they are still in the process of deepening integration and face challenges in harmonizing policies and achieving common objectives (Odijie, 2023). On the other hand, intergovernmental organizations like ASEAN lack comparable legal frameworks and supranational authority, relying more on voluntary cooperation and facing obstacles in achieving consistent policy implementation (Muhibat, 2022).

Overall, these examples illustrate the need for policy frameworks that can effectively address the complexities of food systems, by prioritizing collaboration, increasing awareness to systems thinking, and shared responsibility not only within nations but across borders and regions. Currently, there is a lack of policies and frameworks for sustainable food systems across various geographic scales (e.g., local, regional, national, global) and administrative or organizational levels (e.g., government, community, industry, international organizations) (Zou et al., 2022). Moreover, aligning with the policy cycle framework proposed by Jann and Wegrich (2017), it becomes evident that non-governmental stakeholders are often marginalized or minimally involved in crucial stages of the policymaking process, particularly from agenda setting to decision-making, as highlighted by Kwete et al. (2021). This marginalization exacerbates challenges in policy implementation, where issues are frequently misprioritized, especially in resource-constrained settings like developing countries (Kapiriri, 2012; Kwete et al., 2021). Furthermore, the absence of ‘fit for purpose’ tools and metrics to analyze food environments hinders effective problem identification, intervention selection, progress monitoring, and impact evaluation. To address these shortcomings, opportunities exist for enhancing indicators of food sustainability through innovative approaches such as One Health (Garcia et al., 2020), EcoHealth (Charron, 2011), Sustainable Diets (FAO, 2010), and Planetary Health (Whitmee et al., 2015), while incorporating considerations such as livelihoods, footprints, and access to metrics. Additionally, the prevalence of costly but poorly implemented fine-scale interventions underscores the importance of conducting impact assessments, leveraging metrics to measure policy effectiveness, and incorporating econometrics for comprehensive impact evaluation. Consistent and systematic monitoring and evaluation practices are essential for ensuring the quality and utility of outcomes, thereby fostering the continuous improvement of public policies (OECD, 2021).

### 4.3 Research and data for sustainable development of food systems – placing SDG 17 at the core of the transformative process

Research and data capacities play a pivotal role in enabling a shift from focusing solely on food security to recognizing the broader concept of nutritional security. For instance, with robust data, we can highlight the true costs of unhealthy diets and provide critical evidence on the environmental and social costs of food production and consumption. This allows policymakers and stakeholders to better understand the complexities within food systems, thereby paving the way for interventions that promote access to nutritious foods and sustainable dietary practices. Research and data capacities also play a crucial role in bridging the gap between policy formulation and execution within food systems. By providing evidence-based insights into the effectiveness of existing policies and interventions, research can inform policymaking processes and help identify misplaced or misaligned policies. Through robust data analysis and evaluation, policymakers can gain a deeper understanding of the complex dynamics within food systems and tailor interventions to address specific challenges effectively.

Tackling systemic issues is fundamental to facilitate a more effective transition towards nutrition-sensitive and socially and environmentally sustainable food environments, as well as bridging the gap between policy formulation and execution. The key systemic issues, in the contexts of SDGs, relate to SDG 17: Partnerships for the Goals. More particularly, they are (i) policy and institutional coherence (T 17.14, 17.15), (ii) multi-stakeholder partnerships (T 17.16, 17.17) and (iii) data, monitoring and accountability (T 17.18, 17.19). Food environment transformation requires institutional and policy reforms, while the design and implementation of transformative policies require effective uptake of evidence-based research and recommendations by policymakers (Breitmeier et al., 2021). Hence, we synthesized the research priorities that address these systemic issues (T 17.14–17.19) according to the 5 stages of the policymaking process (Jann and Wegrich, 2017) and presented them in Table 1. These recommendations are research-driven transdisciplinary interventions that are expected to counter the existing policy fragmentation and systemic challenges to making food environments nutrition-sensitive and socially and environmentally sustainable, as detailed in the preceding sections.

In the Agenda Setting phase (Table 1), we focus on defining indicators, developing assessment tools, and building capacities (T 17.18 and 17.19). Moving to the Formulation stage (Table 1), we emphasize the creation of collaborative frameworks, innovative governance and business models, and evidence-based policy formulation (T 17.14, 17.15, 17.16, and 17.17). Throughout Decision-Making, Implementation, and Evaluation/Monitoring stages (Table 1), we introduce tools and frameworks for prioritization, communication, coordination, and impact assessment, maintaining a consistent alignment with SDG 17 targets. The Revision/Correction/Abandonment stage (Table 1) loops back to the initial agenda-setting, emphasizing an iterative and adaptive approach. Throughout the policy making process (Table 1), SDG 17 systemic issues of partnerships, data, and community empowerment are recurrent themes, emphasizing the importance of collaborative and data-driven approaches for sustainable food policies.

Monitoring of progress requires high-quality, timely and reliable data. Statistical capacity building in all countries, particularly developing countries, should be enhanced to harness the full potential of data and technology for sustainable development. Among the first steps towards building statistical capacity is developing a transdisciplinary evidence base. One of the first aims of the evidence base would be to overcome the ambiguity and vagueness of the contexts of food environment, food security, nutrition security, and sustainable and healthy diet. This will pave the way for the development of metrics to assess different drivers of dietary trends and consumption patterns in different settings, measuring diversity and quality of food available, identifying informality and its role in nutrition across the urban gradient and improving the applicability of metrics across different sites.

The implementation of the “true cost” of food, as discussed in Subsection 3.4, would require cross-sectoral collaboration across industries, understanding how trade-offs play out between “winners” and “losers” across the food systems, and the devising of a new system for shared responsibility at all levels. However, the general lack of evidence base for true cost accounting support the notion put forth by the participants regarding the reluctance of policymakers to tax or incentivize food for sustainability. The State of Food and Agriculture 2023 report introduces the concept of true cost accounting (TCA) to assess the hidden environmental, health, and social costs and benefits of agrifood systems (FAO, 2023b). While it presents initial national-level assessments for 154 countries, estimating global hidden costs at 10 trillion 2020 PPP dollars, the findings are still very preliminary and highlight a significant lack of research and data (FAO, 2023b). The report emphasizes the need for innovations in research, data collection, and capacity building, especially in low- and middle-income countries, to make TCA a viable tool for informed decision-making and policy development. Additionally, developing transdisciplinary evidence base such as novel business models and social innovation tools that internalize externalities to food prices fairly and equitably would be helpful to overcome political reluctance, and for policymakers to better understand and devise appropriate tax and incentive schemes for food systems.

Tools that facilitate collaboration and coordination play a pivotal role in bridging the gap between intra- and inter-country dimensions of food systems, enabling stakeholders to exchange knowledge, share best practices, and align strategies to achieve common goals. One such tool would be the establishment of online platforms and knowledge-sharing portals where stakeholders can access and disseminate information related to food systems, fostering collaboration and information exchange. Interdisciplinary workshops and conferences provide another avenue for experts, policymakers, researchers, and practitioners from diverse sectors to convene, discuss challenges, and co-create solutions for sustainable food systems. Additionally, policy coherence mechanisms are essential to ensure alignment between policies at different levels, avoiding conflicting objectives and promoting unified action towards shared goals. Multi-stakeholder task forces and working groups offer opportunities for representatives from government, industry, academia, NGOs, and communities to collaborate on specific food system issues, driving collective action and innovation. Partnership platforms, data-sharing systems, capacity-building programs, cross-sectoral policy dialogues, public-private partnerships (PPPs), and community engagement platforms further enhance collaboration and empower stakeholders to address complex food system challenges collaboratively. These tools collectively contribute to fostering a more sustainable, equitable, and resilient food system by facilitating cooperation and coordination among stakeholders across various levels and dimensions.

**Figure 5:**
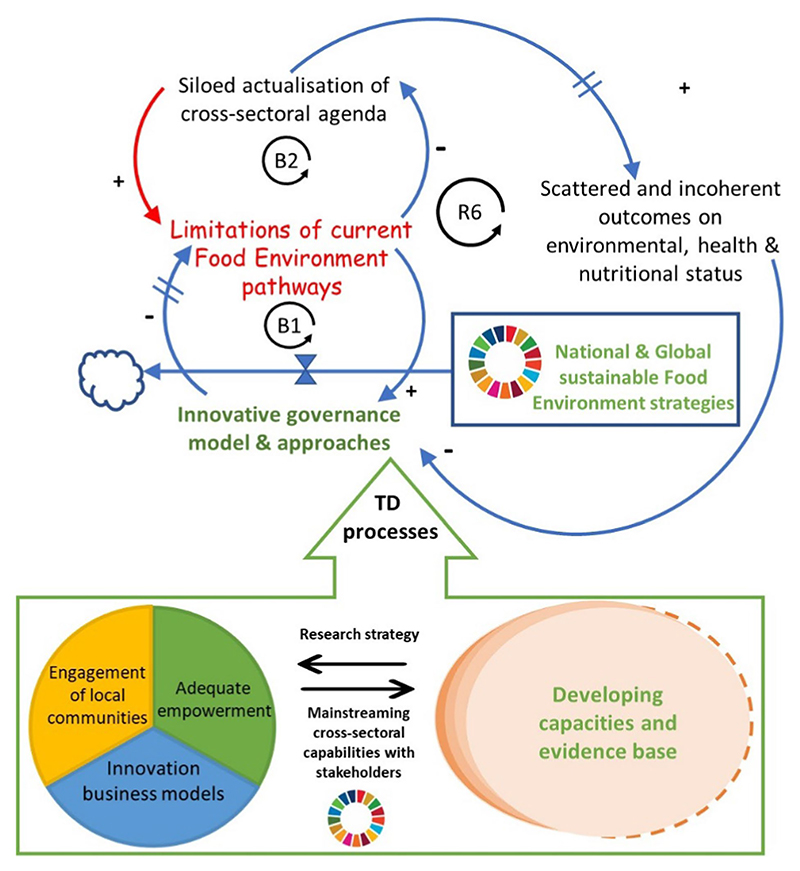
Importance of having a sound causal and intervention hypothesis for path-dependent sustainable trajectories. B1: Balancing loop that seeks to dampen the limitations of current food environment by using novel governance approaches and global frameworks such the SDG targets. Such actions are prone to systemic delays since upstream causes and downstream effects in food environments are complex and occur far in space and time. B2: Balancing loop that describes what actually happens from a realist perspective. To address the FE limitations, siloed actualisation of cross-sectoral agenda overtakes the holistic and system-wide approach. Eventually, there is scattered and incoherent outcomes on environmental, health and nutritional status. R6: Limitations of current Food Environment pathways → Siloed actualization of cross-sectoral agenda → Scattered and incoherent outcomes on environmental, health & nutritional status → Innovative governance a models and approaches → Limitations of current Food Environment pathways. The red arrow represents the unintended and underlying consequences of having a dominant siloed-governance mechanisms to deal with complex ‘Food Environments’ issues that are embedded with socio-technical and socio-ecological evolutionary dynamics. Transdisciplinary processes combined with targeted research strategies have the potential to leverage novel governance needs. The equal sign on the arrows denotes systemic delays.

Embracing SDG 17 – Partnership for The Goal is paramount for advancing the research, data and tools proposed to tackle systemic issues in food systems, as outlined above. SDG 17 serves as a convener and facilitator for all other sixteen goals, directing efforts towards concrete areas of action, including those directly relevant to food environment research (as depicted in Figure 2). However, merely adopting a cross-sectoral agenda is insufficient in unleashing the desired sustainable transformation. Figure 5, employing the ‘Shifting the burden’ system archetype (Wolstenholme, 2003), illustrates the consequences of symptomatic, short-term, and siloed approaches compared to transdisciplinary and trans-sectoral approaches to sustainable food environment transition. When there’s a lack of transboundary foresight, policy actions tend to focus on symptomatic solutions, exacerbating limitations within the food environment system over time. This approach results in scattered and incoherent outcomes on environmental, health, and nutritional status, hindering the establishment of innovative governance. In line with this, collaborative partnerships, as outlined in a biennial UN resolution in 2016 on ‘Towards global partnerships’ (A/RES/70/224, para. 2), involve voluntary and cooperative relationships between various parties, encompassing both public and non-public entities. In these partnerships, all participants agree to collaborate towards a common purpose, sharing risks, responsibilities, resources, and benefits (Basco-Carrera et al., 2021). The implementation of fundamental governance-rooted sustainable measures ought to address the underlying causes of deeply entrenched limitations to have the desirable and overarching effects of the transboundary impact as per SDG 17’s call for countries to align policies and strengthen and streamline cooperation at multi-scales and levels.

While collaboration often promotes creativity, transformation, and positive outcomes (Lahat and Sher-Hadar, 2021), overlooking its less positive dimensions can lead to superficial assessments of impact. Therefore, to operate as a generative mechanism for high impact, other less positive dimensions of collaborative partnerships at the science-policy interface ought to be considered so that Shared Responsibilities can emerge as a systemic response to address food environment issues. Cruz (2023) highlights the complexity of collaborative efforts by identifying key elements such as power relations, trust, goal management, organizational cultures, and leadership. To maximize impact, it is essential to examine factors such as risks, discrepancies, and competing goals in partnerships. Understanding the context, purpose, motivations, and scale of collaboration is essential for diagnosing and addressing diverse approaches and perspectives. By considering both the challenges and potential benefits of collaboration, policymakers and stakeholders can navigate complexities effectively to comprehensively address food environment issues. This integrated approach, grounded in SDG 17 principles, fosters innovative governance and sustainable transformations, leading to positive environmental, health, and nutritional outcomes.

## 5 Conclusion

This paper explores in detail the discussions held during the workshop, identifying key themes and overarching issues that significantly influence both food environments and related health and environmental outcomes. The integrated issues discussed in this paper contribute to setting the goals and research direction of the consortium. The considerable overlap observed among the themes underscores the complexity of these issues and emphasizes the necessity of approaching food environments from a systemic perspective rather than focusing on individual elements.

Our deliberations have underscored the interconnectedness of various factors influencing food environments, emphasizing the need for effective policies and interventions for change to transcend narrow, siloed perspectives. By examining cross-cutting themes such as policy coherence, individual agency, urbanization, and sustainable consumption and production, we have gained a deeper understanding of the multifaceted nature of the challenges confronting efforts to transform our food environments for people and the planet. This holistic view emphasizes the importance of systemic interventions that consider the interplay of social, economic, and environmental factors.

While our discussions have revealed significant barriers hindering progress, including policy mismatches, structural inequalities, and rapid urbanization, they have also highlighted opportunities such as adopting holistic policy frameworks prioritizing collaboration and systems thinking, enhancing statistical capacity building, leveraging innovative tools for policy formulation, and embracing the principles of SDG 17 for meaningful change. By embracing a comprehensive approach that engages stakeholders at all levels and promotes shared responsibility, we can surmount these barriers and foster positive transformations in food systems worldwide. Central to this approach is the recognition of the bidirectional relationship between individuals and their food environments, which underscores the importance of addressing cultural norms, socioeconomic disparities, and structural constraints.

In confronting the global challenge of shaping healthy and sustainable food environments, partnerships and global frameworks play a crucial role. By aligning with Sustainable Development Goal 17 (Partnerships for the Goals) and fostering collaboration across sectors and borders, we can amplify our collective impact and drive progress towards shared objectives. Establishing robust global policy frameworks and international political forums, akin to those addressing transboundary issues such as water and climate change, is imperative for advancing sustainable development goals and ensuring that food environments receive the attention and action they deserve on the global stage.

Our consortium, alongside similar initiatives and social movements, plays a pivotal role in advocating for transformative change on a global scale. Through strategic alliances and partnerships, we can amplify our collective voice and influence policy discussions, ensuring that food environments receive the attention and action they deserve. Global collaboration is needed to advance the development and eventual implementation of proposed tools and interventions. This encompasses data and knowledge-sharing platforms, as well as transboundary policy coherence mechanisms and multi-stakeholder task forces and working groups, together with innovative governance and business models. These efforts are aimed at defining and monitoring sustainable development indicators, developing evidence-based capacities, establishing frameworks for interdisciplinary collaboration, and empowering local communities.

In concluding, while this study provides a comprehensive examination of the challenges and opportunities within global food environments, it also serves as a call to action for continued collaborative efforts. Additional research, policy initiatives, and advocacy are needed to advance our understanding and foster positive change in food systems worldwide. By embracing a transdisciplinary approach and fostering collaborative partnerships, we can work towards creating equitable, resilient, and healthy food systems for present and future generations.

## 6 Limitations

This paper integrates perspectives from five countries with a global lens to analyze food environments, inevitably encountering inherent biases. These perspectives likely include unique challenges and opportunities specific to each region. However, the global lens facilitated by the multinational consortium allows for the identification of commonalities across these diverse contexts. By examining food environments through a sustainability framework, the paper acknowledges the interconnectedness of economic, environmental, and social aspects of food systems. This approach transcends individual biases by exploring broader systemic factors such as production methods, consumption patterns, equity considerations, and policy impacts. Mapping workshop findings against the UNSDGs aims to align the analysis with broader sustainability discourse and global development debates. Additionally, qualitative system dynamics models illustrate complex interlinkages within food environments across different scales and domains.

There are several other limitations to consider, despite the comprehensive approach taken. One significant issue lies in the dynamics of consensus and dissent during qualitative data analysis. Workshop group dynamics are pivotal in generating quality data, yet the emergence of dissonant views may be suppressed by dominant participants. This limitation was overcome by skillful questioning by the moderators and data triangulation. A delicate balance had to be struck in terms of the prominence and involvement of the moderator during the discussion. A review of the transcript found that the input from the moderator constitutes around 10% of the transcript, and is within the range suggested by Hague (1993).

Another bias attributable to the workshop is the lack of face-to-face interactions amongst the participants. However, the virtual platform was necessary at the time, and allowed for better participation as the stakeholders did not need to travel. We refer to the work of Davids et al. (2021) which is based on the Sustainable and Healthy Food System (SHEFS) consortium.^1^ Davids et al. (2021) performed a detailed assessment of the convenience of virtual meetings in terms of carbon footprint, cost and opportunities to develop a learning organization platform. The learning outcomes from SHEFS, especially in terms of encouraging reflexivity, were applied to the current webinar to help moderators deliver optimal facilitation during the interactive session.

In terms of the emphasis on certain topics, the occurrence of the Covid-19 pandemic influenced participants to highlight the importance of establishing governance structures that can respond systemically, especially in times of crisis management. Furthermore, the workshop was largely attended by nutrition advocates; hence there was a tendency to overly criticize large food industries for producing and distributing nutrient-poor foods. While we acknowledge that the food industry plays an important role in post-harvest management and provision of foods enriched with essential nutrients, the problem with growing corporate concentration and power in the global food system may undermine the exploratory nature of this inquiry-based study. Therefore, the involvement of industry players would be included in the next stages of the discussion to ensure a more balanced discussion and consensus building.

We acknowledge that some participants could not get their views across due to poor internet connections. However, this was addressed for some participants by allowing them to post their comments or questions in the chat box.

## Supplementary Material

Supplementary material

## Figures and Tables

**Table 1:** Research-driven transdisciplinary interventions as key priorities to leverage stages of policy process for systemic change in food environments.

Stages of policy process	Outputs and outcomes of transdisciplinary processes to leverage critical realist interventions in food environment transformation	Systemic issues to address as per SDG 17
1. Agenda setting	Defining sustainable development indicators in the context of food environments and developing tools and metrics for baseline assessment Developing capacities and evidence base to support effective problem identification (e.g., spatio-temporal database for food environments)	T 17.18 T 17.19
2. Formulation	Framework for interdisciplinary, intersectoral, multi-regional, global and transboundary collaboration in food policy formulation and implementation Framework to ensure that policymakers identify not only a target audience but also effective communication strategies to reach that audience. Innovative governance model and approaches Innovative business model and approaches to internalise health and environmental externalities	T 17.14 T 17.15 T 17.16 T 17.17
	Framework for effective empowerment and engagement of local communities	T 17.16 T 17.17
	Developing capacities and evidence base to support effective policy formulation (e.g., intervention case study)	T 17.14 T 17.15 T 17.18 T 17.19
3. Decision-making	Tools and metrics for prioritisation of interventions with large scale impact at micro, meso, and macro scales or levels of operation	T 17.18 T 17.19
4. Implementation	Framework for effective dissemination and communication of implementation activities Framework to ensure effective coherence and coordination between the actors involved in implementation efforts across different levels	T 17.14 T 17.15 T 17.16 T 17.17
5. Evaluation/monitoring	Tools and metrics for goal-setting and impact assessment, considering all dimensions of sustainable development at micro, meso, and macro scales or levels of operation	T 17.18 T 17.19
6. Revision/correction/abandonment	Feedback loops linking back to the agenda-setting stage, ensuring continuous improvement and adaptation of strategies across all levels	–

## Data Availability

The raw data supporting the conclusions of this article will be made available by the authors, without undue reservation.

## Footnotes

1
https://shefsglobal.lshtm.ac.uk/
