# Supplementary material for: Transforming food environments: a global lens on challenges and opportunities for achieving healthy and sustainable diets for all
Source: Front Sustain Food Syst. Author manuscript; Available in PMC 2024 Dec 9. (PMC7617158; doi:10.3389/fsufs.2024.1366878)
Supplement: Supplementary material [file EMS197574-supplement-Supplementary_material.DOCX]

Supplementary Material

Emerging Food Environment Themes Through A Global Lens: Commonalities, Challenges and Opportunities For Achieving Nutritional, Social And Environmental Sustainability

EeVon Goh^1*^, Nafiisa Sobratee^2^, Antonio Allegretti^3^, Mallika Sardeshpande^2^, Maysoun Mustafa^1^, Susan Azam-Ali^1^, Rose Omari^4^, Johanna Schott^5^, Vimbayi Grace Petrova Chimonyo^2^, Daniela Weible^5^, George Mutalemwa^3^, Tafadzwanashe Mabhaudhi^2^, Festo Massawe^1^

*** Correspondence:** Dr. Ee Von Goh: eevon.goh@nottingham.edu.my

# Supplementary Figures and Tables

## Supplementary Figures

**Appendix 1**: Overview of The Five Countries

Sources: GDP per capita, 2021 from <https://data.worldbank.org/indicator/NY.GDP.PCAP.CD>. Percentage of urban population from <https://data.worldbank.org/indicator/SP.URB.TOTL.IN.ZS>. Adult overweight and obesity, children and adolescent from 5 to 19 years with obesity and stunting in children under 5 from WHO Global Health Observatory <https://apps.who.int/gho/data/>. Prevalence of moderate or severe food insecurity in the population from <https://data.worldbank.org/indicator/SN.ITK.MSFI.ZS>.

## Supplementary Table

**Appendix 2**: Summary of Food Environment Contexts in the Five Selected Countries

| Country | Trends | Policy | Interventions |
| --- | --- | --- | --- |
| Tanzania | Prevalence of food poverty.  Carbohydrate-heavy, low diversity, low nutrient food consumption.  Liberalisation leading to inflation. | Agrocentric policy promoting food production.  Price stabilisation funds.  Supply chain infrastructure. | Improving information on processed products.  Understanding dietary trends and drivers at household and community level. |
| Germany | Obesity & food poverty are challenging.  Fruit & vegetable consumption has reduced, meat consumption increased.  A slow move to plant-based diets is emerging. | Policy is industry-centric.  Priority is production and marketing.  Nutrition is an individual responsibility and choice. | Better communication of nutritional and environmental information to consumers.  Economic incentives and taxes to promote healthy, socio-ecologically sustainable food. |
| South Africa | Obesity, malnutrition, undernourishment.  Structural inequality and food transitions.  Systemic failure to distribute increased food production and its benefits across populace. | Cross-sectoral policies, plans, and programmes lack coordination.  Scale mismatch between plans and ground implementation. | Improved execution of programmes at national to community level.  Improved access to information, markets, infrastructure, and food. |
| Malaysia | Wheat and wheat-based products, meat consumption and overall food availability on the increase.  Low household fruit and vegetable consumption.  Increased obesity and diet-related non-communicable diseases (NCDs). | National plans for NCD management ineffective.  Agricultural subsidies on rice.  Consumer subsidies on energy dense, low-nutrient foods (flour and cooking oil). | Improved monitoring and surveillance of food and nutrition. Reevaluate agricultural and consumer subsidies to incentivise fruit and vegetable production. Effective advocacy strategies for influencing policy change. |
| Ghana | Gradual shift from unrefined, high fibre carbohydrates to refined, high calory diets, low dietary diversity, low nutrient intake.  Increased obesity and diet-related NCDs and micronutrient deficiencies. | National nutrition policy focusing on both nutrition sensitive and nutrition specific strategies. National NCD control and prevention policy. Legislative instrument for mandatory fortification of wheat flour and vegetable oil. | Nutrition education, promotion of biofortified crops and vegetable production and consumption and development of food-based dietary guideline. |

**Appendix 3:** A Description of relevant SDG targets in relation to food environment issues highlighted during the workshop discussions

| **Goal 2** | |
| --- | --- |
| 2.1 | By 2030, end hunger and ensure access by all people, in particular the poor and people in vulnerable situations, including infants, to safe, nutritious and sufficient food all year round. |
| 2.2 | By 2030, end all forms of malnutrition, including achieving, by 2025, the internationally agreed targets on stunting and wasting in children under 5 years of age, and address the nutritional needs of adolescent girls, pregnant and lactating women and older persons. |
| 2.3 | By 2030, double the agricultural productivity and incomes of small-scale food producers, in particular women, indigenous peoples, family farmers, pastoralists and fishers, including through secure and equal access to land, other productive resources and inputs, knowledge, financial services, markets and opportunities for value addition and non-farm employment. |
| 2.5 | By 2020, maintain the genetic diversity of seeds, cultivated plants and farmed and domesticated animals and their related wild species, including through soundly managed and diversified seed and plant banks at the national, regional and international levels, and promote access to and fair and equitable sharing of benefits arising from the utilization of genetic resources and associated traditional knowledge, as internationally agreed. |
| 2.a | Increase investment, including through enhanced international cooperation, in rural infrastructure, agricultural research and extension services, technology development and plant and livestock gene banks in order to enhance agricultural productive capacity in developing countries, in particular least developed countries. |
| 2.b | Correct and prevent trade restrictions and distortions in world agricultural markets, including through the parallel elimination of all forms of agricultural export subsidies and all export measures with equivalent effect, in accordance with the mandate of the Doha Development Round. |
| 2.c | Adopt measures to ensure the proper functioning of food commodity markets and their derivatives and facilitate timely access to market information, including on food reserves, in order to help limit extreme food price volatility. |
| **Goal 4** | |
| 4.7 | By 2030, ensure that all learners acquire the knowledge and skills needed to promote sustainable development, including, among others, through education for sustainable development and sustainable lifestyles, human rights, gender equality, promotion of a culture of peace and non-violence, global citizenship and appreciation of cultural diversity and of culture’s contribution to sustainable development |
| **Goal 5** | |
| 5.1 | End all forms of discrimination against all women and girls everywhere |
| 5.4 | Recognize and value unpaid care and domestic work through the provision of public services, infrastructure and social protection policies and the promotion of shared responsibility within the household and the family as nationally appropriate |
| 5.5 | Ensure women’s full and effective participation and equal opportunities for leadership at all levels of decision-making in political, economic and public life |
| 5.c | Adopt and strengthen sound policies and enforceable legislation for the promotion of gender equality and the empowerment of all women and girls at all levels |
|  | |
| **Goal 9** | |
| 9.c | Significantly increase access to information and communications technology and strive to provide universal and affordable access to the Internet in least developed countries by 2020 |
| **Goal 10** | |
| 10.2 | By 2030, empower and promote the social, economic and political inclusion of all, irrespective of age, sex, disability, race, ethnicity, origin, religion or economic or other status |
| 10.3 | Ensure equal opportunity and reduce inequalities of outcome, including by eliminating discriminatory laws, policies and practices and promoting appropriate legislation, policies and action in this regard |
| 10.4 | Adopt policies, especially fiscal, wage and social protection policies, and progressively achieve greater equality |
| **Goal 11** | |
| 11.3 | By 2030, enhance inclusive and sustainable urbanization and capacity for participatory, integrated and sustainable human settlement planning and management in all countries |
| 11.4 | Strengthen efforts to protect and safeguard the world’s cultural and natural heritage |
| 11.a | Support positive economic, social and environmental links between urban, peri-urban and rural areas by strengthening national and regional development planning |
| **Goal 12** | |
| 12.1 | Implement the 10-Year Framework of Programmes on Sustainable Consumption and Production Patterns, all countries taking action, with developed countries taking the lead, taking into account the development and capabilities of developing countries |
| 12.6 | Encourage companies, especially large and transnational companies, to adopt sustainable practices and to integrate sustainability information into their reporting cycle |
| 12.8 | By 2030, ensure that people everywhere have the relevant information and awareness for sustainable development and lifestyles in harmony with nature |
| 12.c | Rationalize inefficient fossil-fuel subsidies that encourage wasteful consumption by removing market distortions, in accordance with national circumstances, including by restructuring taxation and phasing out those harmful subsidies, where they exist, to reflect their environmental impacts, taking fully into account the specific needs and conditions of developing countries and minimizing the possible adverse impacts on their development in a manner that protects the poor and the affected communities |
| **Goal 16** | |
| 16.6 | Develop effective, accountable and transparent institutions at all levels |
| 16.7 | Ensure responsive, inclusive, participatory and representative decision-making at all levels |
|  | |
| **Goal 17** | |
| 17.1 | Strengthen domestic resource mobilization, including through international support to developing countries, to improve domestic capacity for tax and other revenue collection |
| 17.6 | Enhance North-South, South-South and triangular regional and international cooperation on and access to science, technology and innovation, and enhance knowledge sharing on mutually agreed terms, including through improved coordination among existing mechanisms, particularly at UN level, and through a global technology facilitation mechanism when agreed |
| 17.9 | Enhance international support for implementing effective and targeted capacity-building in developing countries to support national plans to implement all the Sustainable Development Goals, including through North-South, South-South and triangular cooperation |
| 17.14 | Enhance policy coherence for sustainable development |
| 17.15 | Respect each country’s policy space and leadership to establish and implement policies for poverty eradication and sustainable development |
| 17.16 | Enhance the Global Partnership for Sustainable Development, complemented by multi-stakeholder partnerships that mobilize and share knowledge, expertise, technology and financial resources, to support the achievement of the Sustainable Development Goals in all countries, in particular developing countries |
| 17.17 | Encourage and promote effective public, public-private and civil society partnerships, building on the experience and resourcing strategies of partnerships |
| 17.18 | By 2020, enhance capacity-building support to developing countries, including for least developed countries and small island developing States, to increase significantly the availability of high-quality, timely and reliable data disaggregated by income, gender, age, race, ethnicity, migratory status, disability, geographic location and other characteristics relevant in national contexts |
| 17.19 | By 2030, build on existing initiatives to develop measurements of progress on sustainable development that complement gross domestic product, and support statistical capacity-building in developing countries |
